# The continued effects of COVID-19 on the lives and livelihoods of vegetable farmers in India

**DOI:** 10.1371/journal.pone.0279026

**Published:** 2023-01-03

**Authors:** Sandhya S. Kumar, Pepijn Schreinemachers, Arshad Ahmad Pal, Ravishankar Manickam, Ramakrishnan M. Nair, Ramasamy Srinivasan, Jody Harris

**Affiliations:** 1 World Vegetable Center, South and Central Asia, Patancheru, Telangana, India; 2 World Vegetable Center, East and Southeast Asia, Bangkok, Thailand; 3 World Vegetable Center Headquarters, Tainan, Taiwan; Shandong University of Science and Technology, CHINA

## Abstract

India experienced a rapid rise in COVID-19 infections from March 2021. States imposed varying levels of lockdowns and curfews to curb the spread of the disease. These restrictions severely affected the functioning of food systems. The objective of this study was to analyze how COVID-19 continues to affect agricultural production, food security and household diets of vegetable farmers. A phone-based survey was conducted with 595 vegetable farmers in the states of Andhra Pradesh, Assam, Jharkhand, Karnataka and Odisha, 60% of whom had been interviewed a year earlier. Overall, 60% of farmers experienced decreased vegetable production; over 80% reported a reduction in consumption of at least one food group; and 45% reported some level of food insecurity between May 2020 and May 2021. Farmers who reported decreased staples production, difficulty accessing seeds/seedlings, or reduced their household spending were more likely to report decreased vegetable production. Vegetable consumption was positively associated with receipt of COVID-19 relief benefits, borrowing money, or having home gardens. Farmers who received public agricultural assistance, or had reduced expenses, were more likely to have lower vegetable consumption. Greater severity of food insecurity was associated with farmers belonging to underprivileged social groups, non-Hindus, or those who experienced decrease in livestock production, weather related disruptions or received COVID-19 assistance. This is one of few studies that have conducted a longitudinal assessment of the impacts across multiple waves of COVID-19. COVID-19 is seen to be one among several shocks experienced by farm households, and exacerbated existing issues within agriculture and food security. There is a need for public policy support to strengthen both production and consumption of vegetables.

## Introduction

In the wake of the COVID-19 pandemic in 2020 and in the absence of accurate information on the virus’s transmission, most countries took precautionary measures to curb its spread, which came to be commonly known as ‘lockdown’ measures. It is estimated that due to a combination of the effects of the disease itself and of these lockdown measures, the global economy shrank by five percent and 95 million additional people were pushed into extreme poverty over the first year of the pandemic [1]. India implemented among the most stringent lockdowns, resulting in significant disruptions to food systems [2, 3]. These disruptions stem from physical restrictions to transport and sale of food, labor shortages in agriculture, income losses, disruptions to input supply chains, and the general uncertainty resulting from unclear consumer demand and changing government rules [4, 5].

India’s management of COVID-19 commenced with strict lockdowns across the country at the end of March 2020, bringing most trade and movement of people to a standstill. The initial lockdown was declared for a period of three weeks, however multiple extensions were made until May 30, 2020, after which individual states implemented different policies as per the severity of the COVID-19 situation in their state [6]. While many hailed this as a necessary step to slow the spread of COVID-19, it took a tremendous economic toll as urban centers witnessed an exodus of migrant and informal workers [7, 8], many of whom were forced to return to their villages in the absence of any other work [9]. These restrictions resulted in severe challenges to local food systems, particularly for activities relating to harvesting and marketing of agricultural crops [10].

A few weeks into India’s national lockdown in May 2020, a phone-based survey was conducted across four states with vegetable farmers participating in active projects aimed at promoting vegetable production [11]. The study examined the immediate impacts on sales, incomes and household diets among vegetable farmers as a result of the national lockdown. It found that over 80% of vegetable farmers reported both a decline in selling prices and sales, of which more than half reported a drop in selling prices of over 50%. Furthermore, 62% of households reported some form of disruption to their diet, with the largest reduction in consumption of fruit and non-dairy animal source foods. Notably, 17% of vegetable farmers increased their consumption of vegetables at that time.

Several studies and surveys during the national lockdown reflected similar impacts across India and the region more broadly. A study in Nepal and the state of Gujarat in India on agriculture extension services found that women’s already poor access to such services worsened for over half the respondents due to the COVID-19 lockdown [12]. As a result, nearly half of women farmers, particularly those growing staple crops and vegetables, reported negative impacts on their productivity. Another study in Uttar Pradesh, India’s largest state, found that with increased competition for jobs due to returning migrants as well as the lockdown of local markets limiting vegetables, the incomes of wholesale vegetable farmers and retailers able to sell in local markets increased [13]. The same study also found that retail prices of seasonal vegetables had risen by as much as 40–45%. A study on the impact of the lockdown on agricultural livelihoods and diets across 12 states [14] found that among the 11% of farmers who did not harvest in April-May 2020, 24% cited lockdown-related problems. Of the farmers who had harvested in the same period, only 44% were able to sell crops and 55% of all respondents cited lockdown issues in being able to prepare for the forthcoming sowing season. Almost 80% of households reported reduced wages since lockdown, but impacts of COVID-19 or lockdowns on diets were not assessed. Landless and marginal farmers reported worse outcomes across metrics used, and while most farmers reported receiving higher government food rations, only one third received government agricultural support. Across these studies, COVID-19 is seen to be one among several shocks experienced by farm households, exacerbating existing issues within agriculture and food security.

As restrictions on movement and commerce began to ease within three months of the initial lockdown, food volumes at local markets and wholesale prices began to trend toward pre-lockdown levels [15]. However, the lowering of restrictions on mobility from June 2020 onwards varied across the country, and from month to month. A reduction in daily new cases after a peak in September 2020, led many to believe that the worst of the pandemic had passed. However by April 2021, a second and far more deadly wave had set in [16], this time expanding to rural India due to several factors, including relaxation in norms, public gatherings and festivities, state and village-level elections, and the emergence of more virulent strains of COVID-19 itself [17]. As a result, several states initiated state-level lockdowns and/or curfews of various lengths from April to June 2021 [18].

Sustainability of global food systems has been a growing area of research interest in recent years [19–21]. Having undergone one of the most stringent global lockdowns in 2020, followed by one of the deadliest second waves in 2021, understanding food system disruptions across economic and public health crises among Indian farmers can be instructive for several LMICs where agriculture remains the staple employer for most of the population. With the changing nature of the pandemic, government and market responses evolved in India and across the globe to mitigate the trade-offs between livelihoods, nutrition and health. To better understand how this escalation in contagion and mortality from COVID-19, alongside renewed lockdowns, have affected vegetable farmers in various states of India, this study followed up with respondents from the first survey in 2020 to address the impacts on markets and livelihoods, agricultural production, food security and household diets. The study was also expanded from four to five Indian states by including vegetable farmers in Odisha. This study offers two key contributions to the international literature on the experience of COVID-19 among farmers and of food systems as a whole. First, to the best of the authors’ knowledge, this is one of few studies to follow up with a cohort of vegetable farmers across multiple waves of COVID-19 in India or elsewhere. Second, as an essential player in ensuring healthy diets for the population at large, this study offers some insights into a wide variety of factors that can mitigate and exacerbate the experience of food system shocks for vegetable farmers. This is particularly prescient as COVID-19 continues to affect day-to-day life across much of the globe, including India, and as growing shocks stemming from changing climate conditions increasingly threaten the agricultural sector [22, 23].

## Materials and methods

This study follows up with farmers from a 2020 COVID-19 study [11], which examined the immediate effects of India’s nation-wide lockdowns on vegetable farmers across Andhra Pradesh, Assam, Jharkhand, and Karnataka. The original analytical sample consisted of 448 farmers, who participated in a phone-based survey with 25 questions regarding the changes in their incomes, marketing, and household diets.

The current study expanded its coverage of vegetable farmers to include the state of Odisha to provide a wider lens for understanding how livelihoods and household diets have coped over the past one year of the pandemic. Farmers interviewed in all five states of this study had been engaged in active World Vegetable Center projects in the period of 2019–2021. The two surveys differed between 2020 and 2021, barring select questions on coping measures, inputs access and subjective questions on dietary changes. Therefore, this study primarily analyzes the cross-sectional data collected in 2021, with supplementary analysis to capture some general trends in coping experiences over the past year using both sets of survey data for Andhra Pradesh, Assam, Jharkhand and Karnataka. While the 2020 lockdowns presented a major shock to farmers in the way of restrictions in movement, the deadliness of COVID-19 was more pronouncedly felt in rural India during the second wave, however its effects on farming households have been less understood.

The survey was conducted from May 25, 2021 to June 22, 2021. For the states of Andhra Pradesh, Assam, Jharkhand and Karnataka, the original 448 households that had participated last year were contacted for the survey. In Odisha, 240 farmers were included for this year’s survey. Responses were recorded by enumerators using a customized smartphone application (Akvoflow) to minimize data-entry errors. Enumerators were advised to make three separate attempts to each farmer before submitting them as a non-response, and informed consent was sought at the start of each call. The study posed no significant risk to any participant and was conducted according to the guidelines of the Declaration of Helsinki. The study was exempted from full ethical review by the Institutional Biosafety and Research Ethics Committee (IBREC) of the World Vegetable Center (Exempt 2021–001) on 11 February 2020.

Each interview took 15–20 minutes to complete. Questions included the incidence of COVID-19, agricultural production across seasons, access to markets for sale of produce, household diets, food insecurity and coping measures adopted by households. Questions regarding changes in household diets presented five set responses (e.g., increased, decreased, stopped altogether, no change, did not consume prior). Food insecurity was assessed using the FAO’s food insecurity experience scale (FIES), which presents eight binary questions that progressively assess depth of food insecurity, from worry/anxiety to going without food an entire day [24] during the 12 months between the 2020 and 2021 surveys. For analysis, the responses were tallied across these questions to generate a classification modelled on a food insecurity study in sub-Saharan Africa [25], where we define food insecurity as food secure (score = 0), mild (score≤3), moderate (4≤score<6) and severe (score≥7). Production was recorded as a binary question (e.g.: Did you experience any effects on your vegetable production due to COVID-19 this past year?). For affirmative responses, respondents could select all affected seasons, and for each selection, they had to indicate whether production increased, decreased or stopped altogether. Questions regarding coping measures or non-COVID-19 related factors affecting production, incomes and diets were asked without predefined responses, with enumerators instructed to probe for multiple answers and select the most appropriate responses from a multiple-choice list. Select household and farm data were also collected, including size of farmland under cultivation, main vegetables produced, participants in agriculture work, and social background characteristics (e.g., religion, caste), which are often used as a proxy for socio-economic status in the Indian setting.

From the 2020 sample of 448 farmer respondents, 88 did not participate in the 2021 survey because they either did not consent to be interviewed again or the team was unable to reach them (generally three attempts were made). Five farmers were subsequently dropped from the sample because they did not produce vegetables in the last year, bringing the total analytical sample to 595 farmers. These respondents represent active vegetable farmers in five Indian states and therefore the sample may not be fully representative of all farmers in these states. Back checks were conducted for 10–15% of each enumerator’s surveys for quality assurance.

Descriptive analyses for all the major thematic groups of questions were conducted, including a chi-squared test to compare responses between 2020 and 2021 for common questions relating to access of inputs, sale of vegetables, changes in household diets and coping measures. A binary logit regression model was used to analyze associations between various market, production, public assistance, and household characteristics with decreased vegetable production over the past year. An ordered logit regression model was used to analyze associations between coping measures, government relief, production outcomes, and market-related responses, with changes in household vegetable consumption. An ordered logit regression model was also used to analyze factors associated with food insecurity among respondents who participated in both rounds of the survey, controlling for coping measures, changes in agricultural production, loss in income and sales in 2020, inability to sell vegetables in 2020, household characteristics (e.g., caste and religion), and government assistance. Ordered logit regression was deemed most appropriate for these two models as the variables of interest, household vegetable consumption (none, lower, no change and higher) and food insecurity experience (none, mild, moderate and severe), could be categorized into a sequential order based on the subjective responses from farmers, as shown in Tables 4 and 5. To control for differences between the five states and their respective context, we have also included state dummies with the independent variables. We also tested for attrition bias among the farmers included and excluded across 2020 and 2021 based on select characteristics: gender, household size and land size. A chi-squared test of independence was used for categorical variables (gender) and t-tests used for comparing continuous variables (land and household size). The comparisons did not yield any statistically significant difference, indicating absence of attrition bias (not shown separately). For both the descriptive statistics (chi-squared test) and multivariable regression models, a statistical significance of p <0.05 are discussed in the results of this study.

## Results

### Farm and household characteristics

Of the respondents in the 2021 survey, 23% were women, the majority of whom were from Jharkhand as the WorldVeg project had intentionally selected women farmers in that state (Table 1). Nearly all respondents were farmers producing vegetables on their own land, with the largest mean land size in Karnataka and the smallest in Odisha. Across the five states, 11% of farmers reported that they belong to Scheduled Caste (SC), 22% to Scheduled Tribe (ST), 47% to Other Backward Class (OBC), and 18% belong to the General/Other social groups. (The Constitution of India identifies ST, SC, and OBC communities as being historically disadvantaged compared to the rest of the population in social and economic standing.) Mean land size for vegetable cultivation varied by social group, with those of the SC, ST and OBC categories having significantly lower mean land size under cultivation than those belonging to the General/Other category, by nearly half (not shown separately).

**Table 1:** Select farm and household characteristics, means with the number of observations in parentheses.

	Andhra Pradesh (n = 25)	Assam (n = 131)	Jharkhand (n = 166)	Karnataka (n = 35)	Odisha (n = 238)	Total (n = 595)
Sex (proportion)						
Male	0.92 (23)	0.91 (119)	0.48 (80)	0.94 (33)	0.84 (201)	0.77 (456)
Female	0.08 (2)	0.09 (12)	0.52 (86)	0.06 (2)	0.16 (37)	0.23 (139)
Household size (persons)	4.76	5.27	6.28	6.09	5.42	5.64
Farmers	0.96 (24)	0.98 (128)	0.96 (159)	0.97 (34)	0.94 (223)	0.95 (568)
Mean land (ha)	1.85	1.64	0.60	4.15	0.52	1.07
Mean leased land (ha)	0.97	0.88	0.89	3.16	0.54	0.87
Religion						
Hindu	1.00 (25)	0.68 (89)	0.94 (156)	1.00 (35)	(237)	0.91 (542)
Muslim	0.00	0.32 (42)	0.02 (3)	0.00	0.00	0.08 (45)
Other	0.00	0.00	0.04 (7)	0.00	0.17(1)	0.01 (8)
Social group						
SC	0.00	0.26 (34)	0.10 (17)	0.14 (5)	0.04 (9)	0.11 (65)
ST	0.00	0.04 (5)	0.46 (77)	0.00 (0)	0.19 (46)	0.22 (128)
OBC	0.36 (9)	0.28 (36)	0.38 (63)	0.51 (18)	(156)	0.47 (282)
General/Other	0.48 (12)	0.40 (52)	0.04 (7)	0.29 (10)	0.11 (27)	0.18 (108)
No response/ Unknown	0.16 (4)	0.03 (4)	0.01 (2)	0.06 (2)	0.00	0.02 (12)

SC = Scheduled Caste; ST = Scheduled Tribe; OBC = Other backward class

ha = hectares

**Fig 1:**
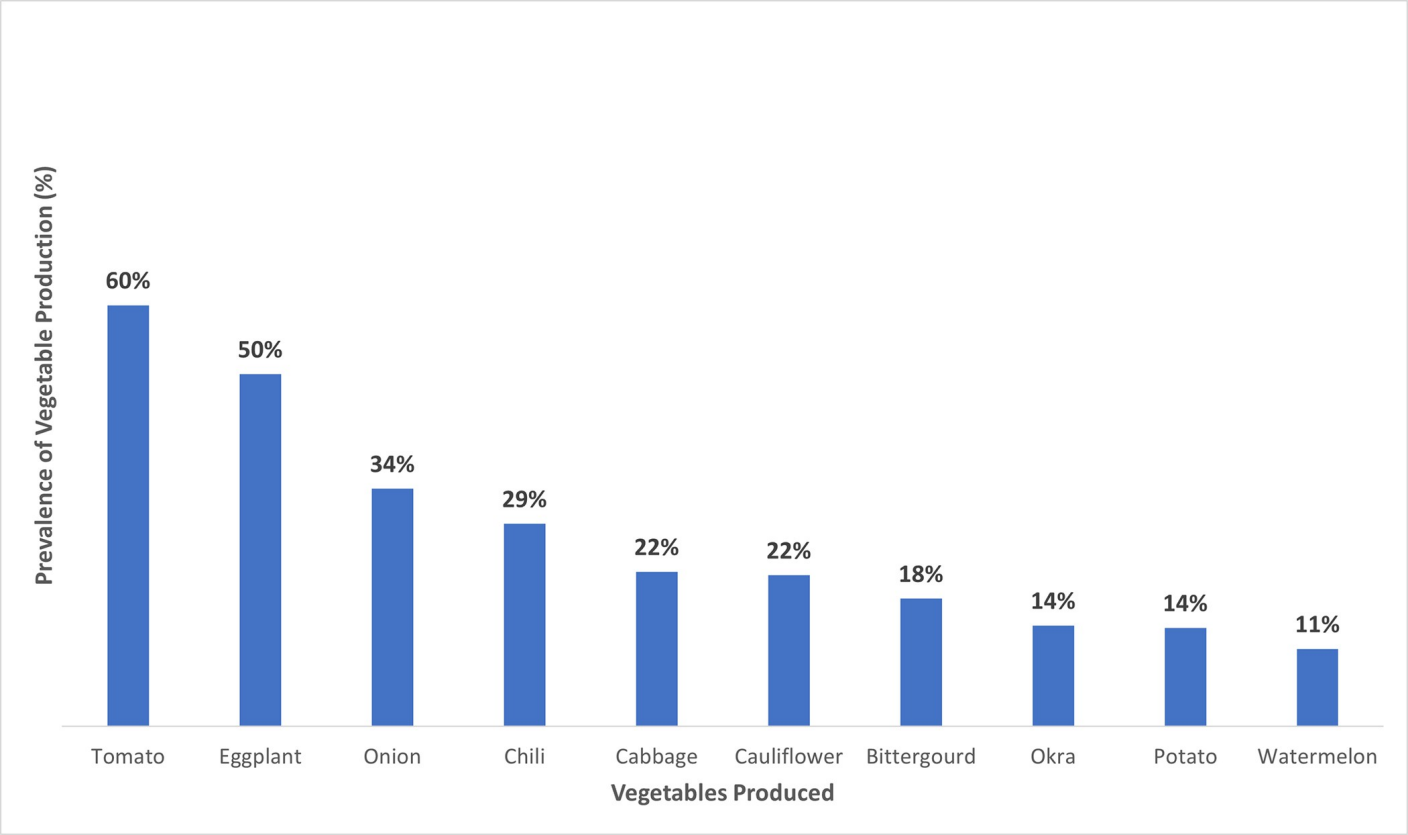
Main vegetable crops produced among surveyed farmers, 2020–2021.

In terms of vegetables produced, 60% of farmers reported tomato production, 50% reported eggplant (*brinjal*), 34% onion and 29% chili (Fig 1). Other commonly grown vegetables included cabbage, cauliflower, bitter gourd, okra, potato and watermelon. With regard to COVID-19, 87% of respondents indicated that no member of their household had been infected with COVID-19, 7% responded that family members had recovered from COVID-19, and 5% reported experiencing symptoms of COVID-19 (e.g., unexplained fever, loss of smell or taste) but were unable to confirm if they were positive for the virus, with three households reporting a COVID-19 death.

### Changes from 2020 to 2021

Table 2 provides a comparison of self-reported challenges faced by farming households in terms of inputs and market access, dietary intake and coping measures compared to pre-pandemic levels. This comparison only accounts for the 357 farmers who participated in both rounds of the survey. Overall, we see that access to inputs and labor was a significantly bigger challenge in 2021 than in 2020 just after the national lockdown, while the ability to sell vegetables significantly improved over the past year.

**Table 2:** Change in market access, diets and coping measures between 2020 and 2021, n = 357.

	2020	2021	χ^2^	p-value
Market access (%):				
Lack of access to inputs	39.7	71.5	7.429	0.006
Lack of access to labor	31.5	46.6	9.251	0.002
Unable to sell vegetables	76.8	57.7	43.124	<0.001
Decrease in dietary intake (%):				
Cereals/starch consumption	17.1	14.0	106.384	<0.001
Vegetable consumption	28.6	22.7	52.316	<0.001
Fruit consumption	55.2	57.4	85.166	<0.001
Legume consumption	31.7	45.7	61.776	<0.001
Meat/egg consumption	53.2	54.3	71.653	<0.001
Dairy consumption	19.9	30.0	6.369	0.012
Household coping measure (%):				
Reduce household expenses	52.1	62.5	51.551	<0.001
Eat more homegrown food	49.9	65.6	1.987	0.159
Eat less	9.0	19.1	37.074	<0.001
Buy cheaper food	13.5	22.1	24.999	<0.001
Borrow money	16.0	34.5	21.815	<0.001
Find other work	13.5	6.4	1.453	0.228
Food aid/support	40.6	82.9	9.519	0.002
Reduce sale prices	27.5	43.4	40.057	<0.001

With regard to household diets, farmers across both survey rounds reported a greater decrease in the consumption of all food groups other than staples and vegetables (i.e., fruit, legumes, meat/egg, dairy) in 2021 than 2020 compared to before COVID-19. Vegetable consumption reduced for 24% of households in 2021, as opposed to 29% in 2020. At the same time, vegetable consumption was higher than pre-COVID levels for about 20% of households in 2021 compared to 11% of the same households in 2020 (not shown separately). Vegetable and cereal consumption are the only two groups for which the majority of respondents cited no change in their consumption compared to before COVID-19. We also see a significant increase in most coping measures, including reducing household expenses, eating less, buying cheaper food, borrowing money, and reducing sales prices, but not in eating more homegrown produce or finding other work.

### Production and markets in 2021

Of the full sample of 595 farmers in 2021, 66% reported that their vegetable production had been affected. In comparison, only 27% and 14% reported that the production of staple grains and livestock had been affected, respectively. Given the variation and severity of the COVID-19 pandemic in India in 2021, we attempted to assess how production was affected in each of three agricultural growing seasons. Among affected vegetable farmers, 90% of those growing vegetables in the *kharif* (wet summer) season of 2020 reported a decline in vegetable production, as did 76% of farmers producing vegetables during the r*abi* (dry winter) season and 93% of those producing vegetables during the summer season as the second wave of the pandemic spread.

Access to inputs was a prevalent challenge among surveyed respondents, with 82% of farmers indicating one or more problems with inputs. Over half of the respondents reported problems in accessing fertilizers and pesticides compared to before COVID-19. Hiring agricultural labor was also a critical challenge for 43%, followed by vegetable seeds/seedlings for 40% of farmers.

As Table 2 shows, the ability to sell vegetables has significantly improved since last year, however 58% of farmers were still unable to sell their vegetable produce over the past year. The resulting unsold vegetables were generally eaten within the household (74% of respondents), distributed to others (66%) or fed to livestock (64%).

Results of the binary logistic regression model (Table 3) help to disentangle some of the factors associated with declined vegetable production over the past year. We find that farmers who reported decreased staple crop production over the past year, were more likely to have decreased vegetable production, as well as those who reported having more difficulty than before COVID-19 in accessing vegetable seeds or seedlings. Farm households not employed under the government’s public works program called the Mahatma Gandhi National Rural Employment Guarantee Act (MGNREGA), and those that reduced household expenses were more likely to have decreased vegetable production. Ability to sell vegetables, having no problems accessing inputs, and being in Assam, Karnataka or Odisha were all more associated with not experiencing decreased production. The sex of the respondent, their social group, and incidence of COVID-19 had no significant association with decreased vegetable production.

**Table 3:** Factors associated with decreased vegetable production 2020–2021.

		n	%	OR	95% confidence interval		p-value
					Lower bound	Upper bound	
Health and Household	Female farmer (1 = yes)	139	72.7	1.11	0.62	1.98	0.721
	*Social group*						
	Others (reference group)	120	46.7	1.00			
	OBC	282	61.4	1.20	0.66	2.17	0.550
	ST	128	67.2	1.10	0.52	2.31	0.805
	SC	65	61.5	1.11	0.48	2.55	0.805
	COVID-19 positive (1 = yes)	49	69.4	2.05	0.92	4.60	0.081
	Healthcare costs (1 = yes)	131	61.1	0.77	0.42	1.40	0.394
Agriculture and Production	Farm is main income source (1 = yes)	509	62.1	1.02	0.56	1.86	0.953
	Total land size (acre)	595	*	1.00	0.96	1.03	0.819
	Decreased staple production (1 = yes)	157	77.1	2.08	1.21	3.58	0.008
	Decreased livestock production (1 = yes)	83	73.5	1.32	0.67	2.62	0.424
	Able to sell vegetable (1 = yes)	267	43.1	0.39	0.26	0.61	<0.001
	Difficulty accessing vegetable seed or seedlings (1 = yes)	236	77.5	2.07	1.27	3.39	0.004
	Difficulty accessing fertilizers (1 = yes)	303	63.0	0.69	0.40	1.19	0.180
	Difficulty accessing pesticides (1 = yes)	318	66.4	1.04	0.63	1.74	0.866
	Difficulty accessing labor (1 = yes)	255	62.4	0.96	0.52	1.76	0.894
	No input problems (1 = yes)	98	41.8	0.30	0.13	0.68	0.004
Income Shocks	Weather-related disruption (1 = yes)	170	55.9	0.81	0.50	1.31	0.388
	Disease/pest in crops (1 = yes)	295	62.4	1.53	0.96	2.44	0.074
	Loss of off-farm employment (1 = yes)	102	74.5	1.74	0.91	3.35	0.094
	Return of migrant workers (1 = yes)	64	64.1	1.26	0.61	2.58	0.530
	Loss of MGNREGA (1 = yes)	89	73.0	2.29	1.17	4.46	0.015
Coping and aid measures	Purchased fewer farm inputs (1 = yes)	127	63.0	1.18	0.67	2.08	0.567
	Reduced sale prices (1 = yes)	208	66.8	0.80	0.49	1.3	0.365
	Reduced household spending (1 = yes)	328	70.7	2.25	1.39	3.64	0.001
	Home gardening (1 = yes)	329	59.3	1.00	0.61	1.63	1.000
	Borrowed money (1 = yes)	255	65.5	1.39	0.87	2.23	0.174
	Sold farm animals (1 = yes)	73	74.0	1.03	0.49	2.19	0.929
	Worked harder/longer hours (1 = yes)	78	59.0	1.18	0.60	2.30	0.635
	Government aid for agriculture (1 = yes)	173	61.9	1.29	0.81	2.06	0.289
State	Andhra Pradesh (reference group)	25	84.0	1.00			
	Assam	131	41.2	0.06	0.01	0.24	<0.001
	Jharkhand	166	83.1	0.43	0.10	1.88	0.261
	Karnataka	35	65.7	0.17	0.04	0.80	0.025
	Odisha	238	50.0	0.06	0.01	0.23	<0.001
	Total	595	59.7				

* Continuous variable

SC = Scheduled Caste; ST = Scheduled Tribe; OBC = Other backward class

While COVID-19 created several economic and health challenges, it was certainly not the only challenge for vegetable farmers (Table 3). Nearly half of all respondents reported problems with pests and disease in crops as negatively impacting household income. Weather-related disruptions were also reported by 29% of respondents as bearing negatively on income. Other reported factors adversely affecting income, and which could be related to COVID-19, included high healthcare expenses (cited by 22% of respondents), loss of off-farm work (17%), and return of migrant family members (11%). Nearly 15% of all respondents also reported negative effects of not being able to avail employment under MGNREGA, which guarantees 100 days of work to rural Indians at fixed daily wages.

### Diets and food insecurity in 2021

**Fig 2:**
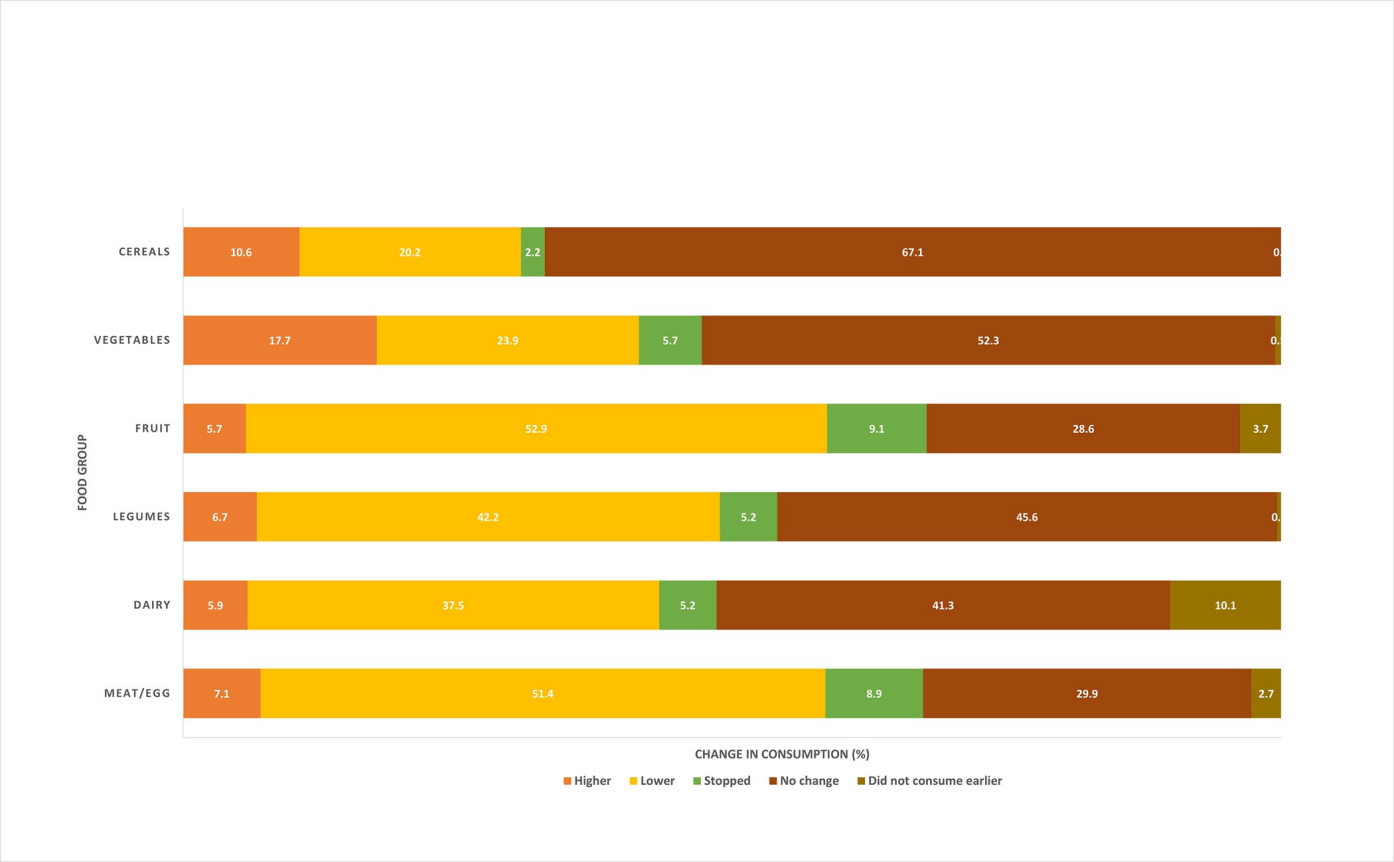
Self-reported changes in household diets since COVID-19.

Nearly 30% of farmers across the five states reported either reduced vegetable consumption or having stopped consuming vegetables altogether as compared to before the pandemic (Fig 2). At the same time, approximately 18% of farmers in 2021 reported increased vegetable consumption compared to before COVID-19. Fruit and non-vegetarian foods (including egg) were reported by over half of respondents as being less consumed than before the pandemic or stopped altogether.

To better understand the factors associated with higher or lower vegetable consumption in households in 2021 compared to pre-pandemic levels, Table 4 shows the results of an ordered logit analysis on vegetable consumption. Decreased vegetable production, reduced household expenses, availing of Integrated Child Development Services (ICDS), and receiving assistance under national or state-level agricultural schemes were all significantly associated with lower levels of vegetable consumption. Factors that were significantly associated with higher levels of vegetable consumption include experiencing weather-related disruptions, disease/pest in crops, loss of off-farm employment, having healthcare costs, receiving COVID-19 relief from a government scheme, home gardening and borrowing money. Farm households in Jharkhand and Odisha were more likely to report lower vegetable consumption compared to Andhra Pradesh.

**Table 4:** Factors associated with vegetable consumption during COVID-19.

							95% Confidence Interval		
	n	None	Lower	No change	Higher	OR	Lower bound	Upper bound	p
Female farmer (1 = Yes)	139	13.0	27.3	42.5	17.3	1.06	0.68	1.63	0.806
Social group									
General/Others	120	1.7	5.8	79.2	13.3	1.00 (reference)			
Other Backward Classes	282	2.5	30.5	54.3	12.8	0.84	0.52	1.37	0.491
Scheduled Tribes	128	21.9	25.8	29.7	22.7	0.70	0.38	1.29	0.249
Scheduled Castes	65	0.0	24.6	38.5	36.9	1.26	0.64	2.48	0.505
Household size	595	*	*	*	*	1.06	0.99	1.15	0.107
Incidence of COVID-19 (1 = yes)	49	2.0	26.5	24.5	46.9	1.88	0.95	3.73	0.071
Farming income source (1 = yes)	509	7.3	26.1	50.1	16.5	0.60	0.36	1.00	0.050
Decreased veg production (1 = yes)	355	9.6	25.9	48.2	16.3	0.60	0.40	0.88	0.010
Decreased cereal production (1 = yes)	157	1.3	31.9	45.2	21.7	0.82	0.54	1.26	0.361
Decreased livestock production in any season (1 = yes)	83	0.0	50.6	39.8	9.6	0.65	0.38	1.11	0.114
Generally able to sell vegetables (1 = yes)	267	8.6	24.0	44.9	22.5	0.75	0.52	1.10	0.137
Weather-related disruption (1 = yes)	170	0.6	22.9	50.0	26.5	1.57	1.03	2.38	0.034
Disease/pest in crops (1 = yes)	295	1.4	22.4	54.9	21.4	1.85	1.28	2.69	0.001
Loss of off-farm employment (1 = yes)	102	1.0	34.3	38.2	26.5	1.80	1.08	3.02	0.025
Healthcare costs (1 = yes)	131	2.3	24.4	42.8	30.5	1.84	1.13	3.01	0.015
Loss of MGNREGA employment (1 = yes)	89	2.3	40.5	48.3	9.0	0.65	0.39	1.07	0.090
*Measures taken to cope with household diet*									
None (1 = yes)	34	2.9	8.8	73.5	14.7	1.72	0.74	3.97	0.205
Reduced food intake (1 = yes)	85	1.2	38.8	37.7	22.4	0.97	0.54	1.72	0.914
Reduced expenses (1 = yes)	436	6.7	28.4	47.0	17.9	0.50	0.31	0.81	0.005
Ate more home-grown food (1 = yes)	410	2.4	25.1	53.7	18.8	1.13	0.72	1.77	0.584
Purchased cheaper food (1 = yes)	178	1.7	37.1	41.6	19.7	1.00	0.64	1.55	0.983
Took on more off-farm work (1 = yes)	37	5.4	18.9	70.3	5.4	0.75	0.36	1.54	0.429
Borrowed money (1 = yes)	201	3.0	28.4	58.2	10.5	0.66	0.37	1.18	0.164
*Access to public food and nutrition programs*									
None (1 = yes)	72	0.0	11.1	72.2	16.7	0.71	0.18	2.77	0.619
Public Distribution System (1 = yes)	516	7.2	25.8	49.0	18.0	1.05	0.29	3.83	0.936
Integrated Child Development Services (1 = yes)	75	17.3	26.7	38.7	17.3	0.54	0.32	0.92	0.024
COVID-19 relief (1 = yes)	138	2.2	24.6	55.8	17.4	1.79	1.09	2.91	0.020
Purchased fewer farm inputs (1 = yes)	127	0.0	21.3	60.6	18.1	1.10	0.68	1.78	0.701
Reduced sale prices (1 = yes)	208	4.3	22.1	54.3	19.2	1.46	0.98	2.17	0.063
Reduced household spending (1 = yes)	328	9.5	22.9	50.0	17.7	0.76	0.50	1.16	0.207
Home gardening (1 = yes)	329	0.9	23.4	54.4	21.3	2.06	1.35	3.13	0.001
Borrowed money (1 = yes)	255	2.8	29.8	52.6	14.9	1.85	1.03	3.34	0.040
Sold farm animals (1 = yes)	73	0.0	32.9	60.3	6.9	1.06	0.60	1.86	0.852
Sold other assets (1 = yes)	30	6.7	46.7	33.3	13.3	0.83	0.36	1.91	0.656
Worked harder/longer hours (1 = yes)	78	3.9	43.6	35.9	16.7	0.80	0.46	1.39	0.433
Received government agriculture assistance (1 = yes)	173	13.9	27.8	49.7	8.7	0.62	0.42	0.91	0.014
*State*									
Andhra Pradesh	25	0.0	20.0	76.0	4.0	1.00 (reference)			
Assam	131	0.0	2.3	68.7	29.0	1.40	0.52	3.80	0.504
Jharkhand	166	18.7	24.7	36.8	19.9	0.29	0.11	0.80	0.016
Karnataka	35	2.9	8.6	88.6	0.0	0.61	0.21	1.77	0.364
Odisha	238	2.1	37.8	46.2	13.9	0.32	0.12	0.81	0.017
**Total**	595	6.2	23.9	52.3	17.7				

Odds ratio values above 1.0 indicate higher vegetable consumption, and odds ratio values below 1.0 indicate lower vegetable consumption, as compared to the reference group.

* Continuous variable

**Fig 3:**
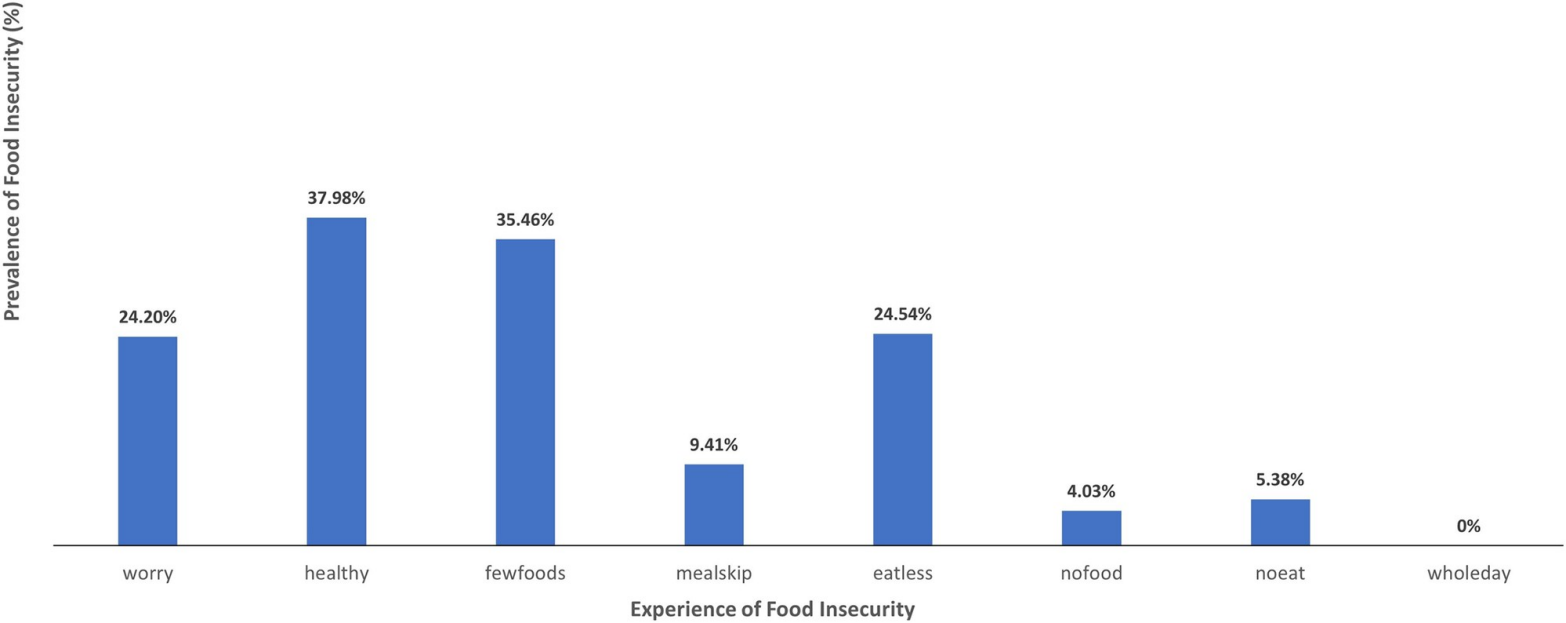
Self-reported experiences along the food insecurity experience (FIES) scale.

In terms of food insecurity, Fig 3 shows that approximately 24% of all farmers had been worried that they would not have enough food to eat during the preceding 12 months. Respectively 38% and 35% of farmers responded that they felt they were unable to eat healthy/nutritious foods and ate only a few kinds of foods. Far fewer farmers cited instances of skipping a meal (9%), their household running out of food (4%) and feeling hungry but not eating (5%), however 25% reported that they had eaten less than they thought they should over the past 12 months.

While gender and social background did not appear to have any significant association with vegetable consumption, in a separate ordered logit model, we examined factors associated with mild, medium and severe scores of food insecurity experience (Table 5) among the 357 farmers who participated in both rounds of this study. This analysis incorporated factors relating to the initial shock of 2020 to livelihoods and diets, as well as accounting for factors in the intervening twelve months. The results show that belonging to a non-General/Others social group and being non-Hindu were significantly associated with more severe levels of food insecurity. Other significant factors associated with severe food insecurity are decreased livestock production, weather-related disruptions, and receipt of COVID-19 assistance from the government.

**Table 5:** Factors associated with food insecurity among farmers across both survey rounds.

		Level of food insecurity								
	n	None		Mild		Moderate	Severe	OR	Std. Err.	p
Sex (1 = female)	102	50.00		42.20		7.84	0.00	0.79	0.245	0.451
*Caste*										
Gen/Others	93	72.04		20.40		6.45	1.08	(1.00 reference)		
SC	126	56.35		32.50		9.52	1.59	4.45	2.018	0.001
ST	82	26.83		67.10		4.88	1.22	9.61	5.129	<0.001
OBC	56	66.07		21.40		7.14	5.36	6.59	3.829	0.001
Religion (1 = non-Hindu)	52	61.54		28.90		9.62	0.00	2.57	1.191	0.042
COVID Status (1 = yes)	33	66.67		24.20		6.06	3.03	0.60	0.313	0.326
Household size	357	*		*		*	*	0.97	0.051	0.519
Veg production disruption in 2020 (1 = yes)	305	55.08		36.70		6.56	1.64	0.77	0.321	0.536
*Farm income in 2020*:										
Increased	20	75.00		25.00		0	0.00	(1.00 reference)		
No change	127	57.48		36.20		4.72	1.57	1.49	1.115	0.591
Decreased	210	51.9		36.20		9.52	2.38	2.75	2.141	0.194
Unable to sell veg in 2020 (1 = yes)	274	57.3		33.20		7.30	2.19	0.53	0.316	0.286
*Loss in Sales in 2020*:										
Low	62	46.77		46.80		4.84	1.61	(1.00 reference)		
Half/Moderate	164	62.80		29.90		6.10	1.22	0.76	0.276	0.445
High	131	49.62		37.40		9.92	3.05	0.90	0.479	0.847
*Coping Measures in 2020*:										
No mitigation (1 = yes)	66	54.55		30.30		10.61	4.55	2.59	1.295	0.056
Produce Less (1 = yes)	59	71.19		22.00		3.39	3.39	0.36	0.150	0.014
Store More (1 = yes)	16	68.75		25.00		6.25	0.00	1.72	1.182	0.431
Find new markets (1 = yes)	190	55.79		37.90		4.74	1.58	1.27	0.469	0.511
Reduce prices (1 = yes)	98	54.08		39.80		5.10	1.02	0.60	0.222	0.167
Eat own produce (1 = yes)	65	75.38		21.50		1.54	1.54	0.57	0.230	0.162
Adapt crop choice (1 = yes)	10	30.00		50.00		20.00	0.00	4.05	3.086	0.067
Find other employment (1 = yes)	48	64.58		27.10		6.25	2.08	1.61	0.824	0.348
Borrow money (1 = yes)	57	45.61		49.10		5.26	0.00	0.76	0.364	0.570
Decreased vegetable production in 2021(1 = yes)	236	50.42		38.10		8.47	2.97	1.11	0.365	0.741
Decreased cereals production in 2021 (1 = yes)	100	61.00		26.00		7.00	6.00	0.85	0.284	0.625
Decreased livestock production in 2021 (1 = yes)	25	24.00		40.00		16.00	20.00	3.54	1.714	0.009
Weather-related disruption in 2021 (1 = yes)	119	47.06		37.00		11.76	4.20	2.87	0.853	<0.001
Disease/pest in crops in 2021 (1 = yes)	166	59.64		34.30		4.22	1.81	0.41	0.119	0.002
Healthcare costs in 2021(1 = yes)	78	44.87		46.20		6.41	2.56	1.39	0.601	0.445
Diet changed 2020 (1 = yes)	227	44.93		44.90		8.81	1.32	1.71	0.626	0.141
Public Distribution System	291	51.2		38.80		7.56	2.41	1.70	0.731	0.214
ICDS	28	53.57		42.90		0.00	3.57	0.62	0.282	0.291
Mid-day Meal (MDM)	29	13.79		75.90		3.45	6.90	2.16	0.985	0.091
COVID-19 Aid	39	20.51		53.90		23.08	2.56	6.95	3.818	<0.001
**State**										
Andhra Pradesh	25	52.00	24.00		12.00		12.00	(1.00 reference)		
Assam	131	69.47		21.40		7.63	1.53	0.09	0.068	0.001
Jharkhand	166	38.55		54.20		6.63	0.60	0.12	0.088	0.004
Karnataka	35	82.86		8.57		5.71	2.86	0.10	0.092	0.014
Total	**357**	55.18		35.60		7.28	1.96			

Odds ratio values above 1.0 indicate more severe food insecurity, and odds ratio values below 1.0 milder levels of food insecurity, as compared to the reference group.

## Discussion

The findings of this study extend the evidence base of other studies on the long shadow cast by the COVID-19 pandemic on agriculture, food security and nutrition. The changing dynamic of COVID-19 itself and its particularly acute effect in the second wave on the health of rural Indian communities, prompted this follow-up study to examine how direct illness as well as changing lockdown policy and market conditions affected livelihoods and diets of vegetable farming households. This study explored key changes between farmers’ experiences in the initial lockdown of May 2020 and during the second wave of COVID-19 in May-June 2021, finding that challenges in accessing inputs had increased, however ability to sell vegetables had improved. A considerable number of farmers reported decreased vegetable production between 2020 and 2021, and our analysis finds that access to inputs, gaps in public programs, and coping measures such as reduced household spending, are significantly associated with this reduction.

Vegetable consumption increased for households in 2021 compared to 2020, with 18% of households seeing greater consumption while 24% saw a decrease and over half saw no change. The food groups most severely affected in both years were fruits and meat/fish/egg, which along with vegetables are particularly nutrient-dense and therefore important for health. Our analysis finds that higher vegetable consumption was positively associated with those reporting a home garden, borrowing money, and disruptions due to weather, disease/pest and loss of off-farm employment. Food insecurity was also analyzed among farmers who were part of both rounds of surveys, which found that caste, being non-Hindu, receiving COVID-19 assistance, decreased livestock production and weather-related disruptions were significantly associated with more severe food insecurity. Reporting to produce less as a coping measure in 2020 and incidence of disease/pest in 2021 were found to be associated with lower levels of food insecurity.

With the vast majority (87%) of respondents citing no incidence of COVID-19 in their household, we found no significant association between COVID-19 infection and vegetable production or consumption. Some respondents may have had reservations reporting COVID-19 infections in their family either due to ongoing stigma and fear in declaring their status [26], or because COVID-19 testing was not widely available in rural areas of India [27]. However, if we look at those who reported having healthcare expenses, we see that while there was no association with decreased vegetable production, there was a positive association with improved vegetable consumption. One possible explanation for this association is that health-seeking behavior may be indicative of socioeconomic characteristics [28, 29]. Specifically, those seeking healthcare services may already have either greater awareness of the impact of their lifestyle decisions, such as diets, or better means to eat more nutritious foods. A second explanation is that given the growing rate of non-communicable diseases and their associated risk factors in rural India, many of which have been linked to diets [30, 31], those who are reporting healthcare expenses may have medical recommendations to adhere to healthier diets.

The agriculture sector has shown considerable resilience despite the COVID-19 pandemic. Even with a complete national lockdown in May 2020, which saw the volume of agricultural goods in local markets plummet by over 60%, within three months these levels fully rebounded [15]. Farmers in this study reported a significant increase in their ability to sell vegetables compared to 2020, most likely due to a combination of the easing of movement restrictions, improved access to markets despite a second wave of the pandemic in 2021, and their own adaptation over time. However, access to inputs has grown as a hurdle compared to last year, most likely because most inputs had already been purchased prior to last year’s national lockdown. Difficulty in access to inputs, particularly vegetable seeds/seedlings that are produced seasonally, was associated with decreased vegetable production in this study, while no difficulty in any input access was associated with increased vegetable production. Access to inputs was a challenge for farmers in India due to the lack of movement of goods, which hit states experiencing low growth particularly hard [32]. In a separate study, 55% farmers reported difficulty in planning for the upcoming sowing season, with 34% citing the high cost of inputs and 20% indicating the unavailability of inputs [14]. A more recent nationally representative study found that medium and large farmers reported were more significantly likely to report no change in inputs compared to small/marginal farmers [33]. The present study found that the greatest percentage of farmers reported decreased production in the season immediately following last year’s lockdown, *Kharif* season 2020, thus corroborating the challenges raised in other studies with input access.

Other income related factors that this study found were associated with decreased vegetable production are the lack of employment/benefits through MGNREGA, as well as reduced household spending. A recent study found that under the national COVID relief packages, 800,000 additional MGNREGA job cards were issued and activity increased from 59% to 62%, but on average households worked 21 out of the eligible 100 days; Odisha was the only state where days worked increased [34]. Some have argued that social welfare programs such as MGNREGA have provided income protection to vulnerable households in earlier crises, such as the financial crisis of 2008, however they remain underfunded [35]. While the present study did not further explore the uses or investment of MGNREGA earnings, it is possible that these earnings support agriculture-related investments, in the absence of which farmers are unable to purchase necessary inputs or hire labor to prepare their land for the coming season. Similarly, in the absence of MGNREGA earnings, households may have to reduce overall household spending, which would include expenditure for agricultural activities. This pathway would merit further study in the future, particularly since such programs are intended to buffer farmers against external shocks.

Food security and diet diversity also remain compromised for many of the farming households surveyed, with half or more of respondents reporting that their consumption of several food groups, including dairy, meat/egg, and fruit is still lower than pre-pandemic levels. These findings are supported by other studies that found that household food expenditures declined during the lockdown, particularly for non-staples such as meat, egg, fruit and vegetables [36], with women bearing the greater brunt of reduced consumption. Similar reductions in consumption of nutritious foods have been seen in previous crises in India and elsewhere in Asia [37], and our findings confirm that these foods are often an early casualty of such shocks.

Social safety nets have played a vital role in maintaining diets and livelihoods during the COVID-19 pandemic. The significant increase in access to public food aid and support between 2020 and 2021 observed in this study is corroborated by other studies, which saw sizable jumps in the provision of public programs, such as the Public Distribution System, expanding from 50% to 91% of surveyed households [38]. Our study also shows a significant association between households receiving COVID-19 relief (often cash transfers) and higher vegetable consumption. The central government had launched a USD 23 billion relief package, the *Pradhan Mantri Garib Kalyan Yojana* (PMGKY), to mitigate the shocks resulting from COVID-19 by scaling up existing programs [39]. This included free grains for vulnerable households (*Pradhan Mantri Garib Kalyan Anna Yojana*, PMGKAY), employment for returning migrants in select states (*Garib Kalyan Rojgar Abhiyaan*, GKRA), a wage increment in the existing employment works guarantee program (MGNREGA), a monthly cash transfer during the initial lockdown of 2020 (*Pradhan Mantri Jan Dhan Yojana*, PMJDY), and a conditional cash transfer for buying cooking gas (*Pradhan Mantri Ujjwala Yojana*, PMUY). The implementation of these benefits was left to individual states with varying levels of institutional capacity, yet nearly 85% of rural India benefited from at least one social protection benefit during COVID-19. In fact, delivery of such programs was greater in rural areas than urban locations, a testament to the infrastructure and responsiveness of public schemes across states [39].

While this may have provided some relief for households, in the larger picture, such transfers are estimated to have made up for less than 10% of average income losses between April and May 2020 [40]. Furthermore, the disruption of other essential nutrition programs, particularly for expectant and lactating mothers and young children through the ICDS, and the Mid-Day Meal Scheme for school-going children, may have sustained implications [41]. The current study found that those receiving ICDS benefits were more likely to have poorer vegetable consumption. While there were several court and government orders to keep ICDS services pertaining to nutrition available during the pandemic, the upkeep and delivery of these services faced significant disruption [42]. The negative association our study finds between availing ICDS benefits and poor vegetable consumption may point to the extent of deprivation of the households seeking these benefits during the pandemic, and the broader deficits in quality nutrition and diets.

Beyond the disruption of COVID-19, weather-related disruptions and disease/pest in crops were two factors that showed strong association with both vegetable consumption and food insecurity. This is important to highlight given the growing external shocks to agriculture through climate change that have been underway and are expected to worsen [43]. Other studies have also highlighted that weather and pest-related disruptions were highly prevalent during the COVID-19 pandemic in South Asia, negatively affecting agricultural output and earnings [14, 44]. In the present study, both factors were found to be positively associated with increased vegetable consumption. This may be explained by households resorting to consuming whatever produce is saved, if they have lost a significant share of their vegetables intended for sale. However, this would need further exploration and evidence from future studies.

Several coping measures were found to be significantly associated with vegetable consumption in the present study. Home gardening and borrowing money were found to be positively associated with vegetable consumption. Studies on the impact of home/kitchen and school gardens have till date presented mixed results [45]. However recent work on the impacts of school and kitchen gardens, particularly when working in tandem to support vegetable gardening, have shown promising uptake of knowledge as well as consumption and liking for vegetables among both children and caregivers [46]. This finding is also consistent with the baseline study, which found that households that showed increased vegetable consumption in the aftermath of the national lockdown in India were likely consuming their own produce, therefore improving household nutrition [11].

Caste and religion were two factors that proved to be significantly associated with more severe food insecurity among respondents, though they were not significantly associated with vegetable consumption or production in this study. An earlier multi-state study of rural Indians examined the influence of caste and religion on calorie gaps and also found that households from “low caste” groups or a religious minority were adversely affected in terms of food security, measured by calorie consumption [47]. However, the same study highlights that the intersection of these identities with food security varies by region and the factors that affect poverty do not necessarily affect food security. A recent study on fruit and vegetable consumption between different caste groups attempts to address this by looking at different drivers of consumption, such as income and education, as well as the strength of relationships between these drivers and consumption [48]. It is also important to note that diet patterns vary greatly by social and cultural group preferences and again from region to region in India, which may also explain lower or reduced vegetable consumption. The study goes on to find that differences in fruit and vegetable consumption between “upper” castes and “lower” castes could be explained by differences in income across caste groups, thus recommending short- to medium-term public interventions such as cash transfers and in the long-term to strengthen policies targeting education and employment. While caste and income are strong correlates of fruit and vegetable consumption, other factors such as households headed by females, rural location and relative prices of foods can strongly influence consumption [49]. Considering the economic influence on consumption among more marginalized groups, COVID-19 likely limited their earning potential due to market and mobility restrictions as well as the kinds of work they could do, which continues to be dictated by caste membership in much of rural India. However as much of the work highlights the varying effects of caste and religion along income and consumption levels, further study to understand the intersectionality of different social identities is needed.

While this study provides novel insights on the evolving effects on vegetable farmers, their livelihoods, household diets and the agriculture sector at large, there are some limitations that should be kept in mind in interpreting these findings. First, this study was conducted using convenience sampling of vegetable farmers that are currently or were previously active in WorldVeg programs. Therefore, the findings cannot be generalized as this may not be a representative sample of the farming population within the state or at the national level. Second, as the results of this study are based on self-reported data on experiences over the preceding 12 months, there is the possibility for recall errors, though we kept the questions very simple to minimize such errors. Third, some responses may reflect social desirability bias as respondents are sensitive about disclosing COVID-19 status or household deprivation as it pertains to diets and food insecurity. Despite these limitations, this study is unique in the Indian setting to follow-up with the same group of farmers between the initial lockdown and the second wave of the pandemic.

## Conclusion

COVID-19 has caused extensive upheaval in food systems globally. The initial shock of the pandemic in 2020 led many countries, including India, to impose stringent lockdowns, the effects of which were still being felt one year on. The onset of the second wave in India and its human toll, in both urban and rural India, has been described by some as the greatest tragedy since the country’s independence and partition [50]. This study explored the impacts of the year-long battle with COVID-19 on vegetable farmers across five Indian states, who are an integral part of the national and regional food system. While the easing of market restrictions and mobility have improved the ability of farmers to sell their produce, this study finds that access to essential inputs for agricultural activities remains a challenge. This may have been due to ongoing gaps in the availability of these inputs, but also due to lost incomes which may have rendered them inaccessible for farmers.

While several states and the central government have initiated schemes to support the agricultural sector, ensuring timely delivery of such support may aid farmers in undertaking critical investments and planning for their agricultural activities which are highly time sensitive. The extension or expansion of public programs to support incomes and nutrition, such as MGNREGA, ICDS and COVID-19 relief, may help vulnerable households cope with shocks to their farm incomes and resulting shortages in purchasing power for healthier diets. Vulnerable households, particularly those belonging to historically marginalized communities, require targeted attention during such periods as they may be more likely to be overlooked, and are more likely to already be affected by issues which COVID-19 has exacerbated.

Finally, even when COVID-19 passes, there remain several imminent disruptive factors in the region that will likely continue to impact agriculture and vegetable farmers in particular. Policy interventions and partnerships to evolve agricultural and community practices towards sustainability will be critical for bolstering resilience against future shocks.
